# Infection control in burn patients: are fungal infections underestimated?

**DOI:** 10.1186/1757-7241-17-51

**Published:** 2009-10-09

**Authors:** Manuel F Struck

**Affiliations:** 1 Burn Trauma Center, Bergmannstrost Hospital, Merseburger Str. 165, 06110 Halle (Saale), Germany

## Abstract

With great interest, I read the paper of David J. Dries about recent developments, infection control and outcomes research in the management of burn injuries [1]. I have some comments about an important, however missing, topic in the paragraphs concerning infection control.

## 

Dear Sir,

With great interest, I read the paper of David J. Dries about recent developments, infection control and outcomes research in the management of burn injuries [1]. I have some comments about an important, however missing, topic in the paragraphs concerning infection control.

Infectious complications and sepsis are still the most important reasons of mortality in burn centres. Therefore, not only bacterial infections should be considered as the source of infection. Fungal colonization and infection gain a rising importance in the management of sepsis in burn patients. Burn patients are at the highest risk for mycoses, even more than oncologic and transplant patients [2]. Due to compromised immune defence and large wound surfaces, burn patients are predisposed for acquiring fungal organisms. The broad use of topic and systemic antibiotic agents either as prophylaxis or in case of confirmed bacterial infection further facilitates the development of mycoses.

Recently, a certain dynamic in the epidemiology of fungal organisms has been observed. Non-albicans *Candida *species have been found to be increasingly resistant against common antimycotic substances. Additionally, other species such as *Aspergillus *and Zygomycoses, with an aggressive and invasive growth pattern are more frequently observed. The diagnostic methods to indentify mycoses are still poor and often specific to some organisms. Direct tissue biopsy is performed rarely and mostly in case of a justified suspicion. The growth of fungal cultures is unreliable and associated with considerable latency - sometimes too late for the clinician to initiate antimycotic therapy appropriately. Since burn patients usually present with SIRS symptoms, clinical warning signals may be masked or misleading to bacterial infection. The author correctly highlights the need for a re-evaluation of definitions of SIRS and sepsis, as previously published [3].

Risk factors for acquiring a fungal infection are greater burned total body surface area, increasing age, late surgical excision, central venous catheters, hyperglycaemic episodes, steroid treatment, long-term artificial ventilation and inhalation injury. Mortality of mycotic burn patients is associated with i.v.-antimycotics, the presence of fungaemia, multiple positive cultures and invasion of healthy skin [2,4,5]. Although there exist no randomized controlled trials to initiate a timely antimycotic prophylaxis in burn patients, a lower threshold may decrease the risk of fatal fungal sepsis. Contra-arguments may be the possible development of antimycotic resistances and increasing costs.

Available antimycotic substances such as echinocandins and triazoles show advantages compared to classic imidazol-based azoles and polyenes concerning efficacy, specifity, toxicity profile and patient comfort. Promising results are to be expected by candida-secretoric aspartic proteases (SAPs) inhibitors and calcineurin signaling pathway blockers [6].

However, despite the introduction of new antimycotic substances, some fungal organisms preserving angioinvasive and proteolytic potential, still require radical surgical therapy to provide a chance for survival. The restoration of immune resistance, early surgical therapy and early wound closure gain a key function in limiting the risk of fungal infection in burn patients [2,3,5].

Fungal infections should not be underestimated in modern burn care.

## Abbreviations

SAPs: secretoric aspartic proteases; SIRS: severe inflammatory response syndrome

## Competing interests

The author declares that they have no competing interests.
